# Upholding confidentiality in the preparation and distribution of medication in prisons: implementing recommendations of the European Committee for the Prevention of Torture and Inhuman or Degrading Treatment or Punishment

**DOI:** 10.12688/f1000research.21895.1

**Published:** 2020-02-06

**Authors:** Nguyen Toan Tran, Hans Wolff

**Affiliations:** 1 Australian Centre for Public and Population Health Research, Faculty of Health, University of Technology Sydney, PO Box 123, Sydney, NSW 2007, Australia; 2 Division of Health in Prison, University Hospitals of Geneva, Ch. Du Petit-Bel-Air 2, CH-1225 Chêne-Bourg, Geneva, Switzerland; 3 European Committee for the Prevention of Torture and Inhuman or Degrading Treatment or Punishment (CPT), Council of Europe, Avenue de l'Europe, 67075 Strasbourg, France

**Keywords:** Access to medication, preparation, distribution, detention, prison, confidentiality, autonomy, human rights, CPT, equivalence of care, professional independence.

## Abstract

Confidentiality must be ensured even in the preparation and distribution of medications in detention settings. In this respect, the European Committee for the Prevention of Torture and Inhuman or Degrading Treatment or Punishment found during recent prison visits several instances where prison staff, and at times detainees, dispensed prescribed treatments and supervised their intake. Such a practice compromises medical confidentiality requirements and the establishment of a trusting doctor-patient relationship. To respect medical confidentiality and ensure safety and quality of care, the authors argue that only qualified healthcare personnel should prepare and distribute prescribed medications, all of which require specialized training. They call for robust research that examines the operational barriers and facilitators as well as the respect of human rights related to various approaches to medication preparation, distribution, and intake so that people in detention can access their treatment with safety, confidentiality, autonomy, and dignity.

## Background

The European Committee for the Prevention of Torture and Inhuman or Degrading Treatment or Punishment (CPT) provides a non-judicial preventive mechanism to protect persons deprived of liberty against torture and other forms of ill-treatment ^1^. The CPT visits detention places in the 47 member states of the Council of Europe. The visits include the assessment of medical services in carceral settings to gain insights into the way individuals access healthcare prevention, treatment, and care. The access to healthcare services for individuals experiencing incarceration is essential as this priority population carries a high burden of physical and mental health conditions ^2– 5^. Operating such services in prison must be underpinned by the following guiding principles: timely access to competent medical staff who work independently from the criminal justice system ^6^, equivalence of preventive and curative care services that are free-of-charge to patients (i.e., states bear all the healthcare costs), humanitarian assistance to individuals with increased vulnerabilities (e.g., mothers with children, adolescents), and patient’s right to informed consent and confidentiality ^7^. Failing to respect these principles can result in situations falling within the scope of inhumane and degrading treatment. The objective of this article is to underscore the paramountcy of upholding confidentiality in the preparation and distribution of medication, as highlighted by recent CPT visits.

## Examples of violations

There is a paucity of published peer-reviewed literature on this subject and our sources stem from published CPT reports. Once prescribed, medications require coordinated preparation and distribution for individuals to have timely access to their treatment. In Scotland, the CPT delegation found that medical and prison staff shared the task of distributing medication and supervising its intake ^8^. In Greece, prison staff and detained individuals were acting as orderlies (i.e., they were trained in first aid and selected clinical tasks), who dispensed medication under the supervision of nurses and had access to patient medical records ^9^. In Norway, it was the duty of the custodial officers to distribute prescribed medications despite the daily presence of a nurse ^10^. In Estonia, nurses prepared the medication, and custodial staff ensured its distribution except for psychotropic treatment, which was delivered by nurses ^11^. In Cyprus, several prison officers worked as medical orderlies who distributed medication and accompanied doctors on their rounds, with a few assigned to medical duty during the day or at night ^12^. In the Netherlands, external pharmacies prepared the medication and custodial staff carried out its distribution. For prescribed opioids and psychiatric medication, prison officers also had to check that recipients swallowed them properly (patients reported that they had occasionally received the wrong medication, for example that of their neighbor, due to a lack of attention by the prison officer) ^13^.

## CPT rules and recommendations

Cooperation between healthcare professionals and prison authorities is necessary if it is deemed reasonable and respects the guiding principles mentioned above. However, in the given examples, the distribution of medication, including psychotropic substances and methadone, by prison officers or by incarcerated persons compromised the respect of medical confidentiality—the medication name and its dosage were visible. Such a practice could compromise medical confidentiality requirements and the establishment of a proper doctor-patient relationship.

According to the 1992 CPT report, *there should be appropriate supervision of the pharmacy and the distribution of medicines. Further, the preparation of medicines should always be entrusted to qualified staff (pharmacist/nurse, etc.)* ^7^ In other words, to respect medical confidentiality and ensure safety and quality of care, individuals in prison and custodial staff should not be involved in performing healthcare tasks and only qualified healthcare personnel should prepare and distribute prescribed medications, all of which require specialized training.

We acknowledge the difficulty in following this recommendation, especially in smaller detention facilities, where healthcare professionals are not available 24h/7 when compared to larger and high security facilities. These facilities tend to have the most comprehensive healthcare services and staffing because the patient population is older and present for a longer period ^14^. However, irrespective of the size of the detention centers, people experiencing incarceration use health services more often when compared with the community ^15^. We also understand that ensuring access to medication while respecting confidentiality, complying with prison security requirements, and accounting for fears of theft, trafficking, and misuse, especially of psychoactive medication ^16^, needs a balanced operational approach.

## Recommendations for best practice

The best-case scenario, one that respects medical confidentiality and quality of care while empowering patients in their autonomy, would comprise the following steps: as blister medication increases adherence to treatment ^17^, prescribed medications are left in their blisters (for tablets) and packaged individually for each patient by healthcare staff (such as in the detention facilities of Geneva, Switzerland) or an automated pharmacy preparation system (such as in larger facilities in France); to avoid the confusion of roles between healthcare and prison staff ^18^ and to respect patients’ right to confidentiality ^19^, healthcare professionals—not prison officers or incarcerated individuals—distribute the medications either in-hand (as privately and confidentially as possible) or in individual lockable medication boxes that each user can independently access (such as at La Brenaz facility in Geneva); and when supervised intake is not medically indicated, patients should have the option of taking their treatment in the privacy of their cells.

However, based on the CPT experience, there are limited examples that adhere to this best-case scenario. Therefore, we call for robust operational research that examines the operational barriers and facilitators as well as the respect of human rights related to various approaches to medication preparation, distribution, and intake. Identifying and disseminating best practices that are rights-based will allow people in detention to access their treatment with safety, confidentiality, autonomy, and dignity.

## Data availability

No data are associated with this article.

## Disclaimer

Although HW is a member of the CPT, the opinions expressed in this article reflect those of the authors and are based on CPT reports and recommendations.
