# Supplementary material for: Upholding confidentiality in the preparation and distribution of medication in prisons: implementing recommendations of the European Committee for the Prevention of Torture and Inhuman or Degrading Treatment or Punishment
Source: F1000Res. 2020 Feb 6;9:87. [Version 1] doi: 10.12688/f1000research.21895.1 (PMC10620482; doi:10.12688/f1000research.21895.1)
Supplement: Peer review history

# Reviewer report: Reviewer response for version 1

**DOI:** 10.5256/f1000research.24138.r174975

**Published:** 2023-11-01

**Authors:** Joerg Pont

**Affiliations:** 1 Medizinische Universität Wien, Vienna, Austria

This article is a concise presentation of a much debated issue in many penitentiary health care services. Due to shortage of health care professionals several penitentiary systems resort to non-medical staff or even inmates for dispensing prescibed medication, a practice that clearly is in conflict with the professionally indispensible ethical principle of medical confidentiality and that violates the patients' right to have their medical data kept in medical confidentiality.The article also provides practical recommendations how to overcome this problem.

 For the same reason, the authors may consider also recommending for medically indicated supervised  intake of medication that the supervison should be conducted by health care staff and not by custodial staff and, if presence of custodial staff is deemed necessary, they should stay outside of the room.

 Please correct in the text the year of the 3rd General Report of the CPT in accordance to ref 7, i.e., 1993 not 1992.

Is the topic of the opinion article discussed accurately in the context of the current literature?

Yes

Are arguments sufficiently supported by evidence from the published literature?

Yes

Are all factual statements correct and adequately supported by citations?

Yes

Are the conclusions drawn balanced and justified on the basis of the presented arguments?

Yes

Reviewer Expertise:

Prison health care; medical ethics in prison health care; Internal medicine;

I confirm that I have read this submission and believe that I have an appropriate level of expertise to confirm that it is of an acceptable scientific standard.

# Reviewer report: Reviewer response for version 1

**DOI:** 10.5256/f1000research.24138.r62164

**Published:** 2020-04-17

**Authors:** Nickolas Zaller

**Affiliations:** 1 Department of Health Behavior & Health Education, Fay W. Boozman College of Public Health, University of Arkansas for Medical Sciences, Little Rock, AR, USA

Overall, this is a very well written commentary with some important implications. I do think there could be a few additional considerations incorporated in order to broaden the applicability of the perspective and recommendations. For example, in some parts of the world (e.g. US) correctional facilities contract medical services to a third-party vendor. As such, there needs to be additional coordination between corrections-based staff and non-corrections based medical staff, especially with respect to facilitating medical appointments and medication receipt. With respect to the recommendations for best practice, while the best-case scenario the authors outline is one that all facilities should strive to achieve, it would be nice to see some gradation of compliance with this best-practice in order to make it a bit more practical and/or feasible. For example, if facilities do not have the resources, especially in terms of medical staff to adequately supervise medication, what can be done to mitigate concerns re breaches in confidentiality? It would be good to see recommendations for facilities across a “spectrum” of compliance such that those facilities in poor resourced settings do not feel as though they must undertake an all or nothing approach. Again, if facilities can take measured steps toward achieving the stated best-case scenario, they should be strongly encouraged to do so.

Is the topic of the opinion article discussed accurately in the context of the current literature?

Yes

Are arguments sufficiently supported by evidence from the published literature?

Yes

Are all factual statements correct and adequately supported by citations?

Yes

Are the conclusions drawn balanced and justified on the basis of the presented arguments?

Yes

Reviewer Expertise:

NA

I confirm that I have read this submission and believe that I have an appropriate level of expertise to confirm that it is of an acceptable scientific standard.
